# Combination of Adrenomedullin with Its Binding Protein Accelerates Cutaneous Wound Healing

**DOI:** 10.1371/journal.pone.0120225

**Published:** 2015-03-17

**Authors:** Juan-Pablo Idrovo, Weng-Lang Yang, Asha Jacob, Michael A. Ajakaiye, Cletus Cheyuo, Zhimin Wang, Jose M. Prince, Jeffrey Nicastro, Gene F. Coppa, Ping Wang

**Affiliations:** 1 Department of Surgery, Hofstra North Shore-LIJ School of Medicine, Manhasset, New York, United States of America; 2 Center for Translational Research, Feinstein Institute for Medical Research, Manhasset, New York, United States of America; University of Louisville, UNITED STATES

## Abstract

Cutaneous wound continues to cause significant morbidity and mortality in the setting of diseases such as diabetes and cardiovascular diseases. Despite advances in wound care management, there is still an unmet medical need exists for efficient therapy for cutaneous wound. Combined treatment of adrenomedullin (AM) and its binding protein-1 (AMBP-1) is protective in various disease conditions. To examine the effect of the combination treatment of AM and AMBP-1 on cutaneous wound healing, full-thickness 2.0-cm diameter circular excision wounds were surgically created on the dorsum of rats, saline (vehicle) or AM/AMBP-1 (96/320 μg kg BW) was topically applied to the wound daily and wound size measured. At days 3, 7, and 14, skin samples were collected from the wound sites. AM/AMBP-1 treated group had significantly smaller wound surface area than the vehicle group over the 14-day time course. At day 3, AM/AMBP-1 promoted neutrophil infiltration (MPO), increased cytokine levels (IL-6 and TNF-α), angiogenesis (CD31, VEGF and TGFβ-1) and cell proliferation (Ki67). By day 7 and 14, AM/AMBP-1 treatment decreased MPO, followed by a rapid resolution of inflammation characterized by a decrease in cytokines. At the matured stage, AM/AMBP-1 treatment increased the alpha smooth muscle actin expression (mature blood vessels) and Masson-Trichrome staining (collagen deposition) along the granulation area, and increased MMP-9 and decreased MMP-2 mRNA expressions. TGFβ-1 mRNA levels in AM/AMBP-1 group were 5.3 times lower than those in the vehicle group. AM/AMBP-1 accelerated wound healing by promoting angiogenesis, collagen deposition and remodeling. Treatment also shortened the days to reach plateau for wound closure. Thus, AM/AMBP-1 may be further developed as a therapeutic for cutaneous wound healing.

## Introduction

Cutaneous wounds continue to cause significant morbidity and mortality despite advancements in wound care management. Acute wounds from trauma can become chronic non-healing wounds in the setting of diseases, such as diabetes and cardiovascular diseases [1–3]. The prevalence of chronic wounds currently is up to 6.0 million people per year in the US and 18 million worldwide. An estimated cost of $10 billion is spent annually in the US on wound care associated with diabetic foot, venous stasis and pressure ulcers [4]. Patients could die from complications of chronic wounds such as wound infection, sepsis and septic shock, as well as thromboembolic events from prolonged immobilization [5–7]. Thus, there is an urgent need to develop therapeutics that can promote wound healing.

Wound healing is a complex biological processes initiating with an inflammatory stage proceeding through proliferation and maturation [8]. This process requires a well-coordinated interaction between mediators which are both resident and infiltrating ones. During wound healing after the initial inflammatory responses, new stroma consisted of fibroblasts, macrophages and blood vessels termed granulation tissue is formed. Within these tissues, a large number of growth factors and cytokines are produced which promote tissue healing. Fibroblasts are activated in response to various factors, e.g., TGFβ-1 to induce fibroblast proliferation and migration to the injured site [9]. TGFβ-1 also promotes the production of collagen-rich matrix and contributes to the differentiation of fibroblast to myofibroblast. The myofibroblast acquire contractile capacity and facilitate wound closure [9,10]. Matrix metalloproteinases 2 and 9 also play crucial roles in remodeling the extracellular matrix, leading to wound maturation [9–12]. In chronic wounds, these physiological sequelae of events are disturbed mainly due to the wound environment. Excessive scarring and defects in tissue repair lead to the development of chronic wounds. Dermal fibroblasts are highly involved in these processes and contribute to scarring and impaired wound healing [13].

Adrenomedullin (AM) is a 52-amino acid vasoactive peptide first isolated from human pheochromocytoma cells and acts as a circulating hormone to induce various biological activitiesin a paracrine or autocrine manner. AM is multifunctional in nature and can regulate the proliferation, differentiation, and migration of a number of different cell types as well as exert its regulatory abilities on blood pressure, water, and electrolyte balance [14]. A specific AM binding protein (i.e., AMBP-1) was isolated from human plasma and found to be identical to complement factor H [15,16]. AMBP-1 is a 150-kDa protein which is composed of 20 repetitive domains of short consensus repeats (SCR), with each SCR being about 60 amino acids in length [17]. AMBP-1 can facilitate AM to bind to its receptors and modulate AM biological activity [16]. Moreover, AMBP-1 can prolong the half-life of AM in circulation by protecting it from protease degradation (reviewed by [18]). AM in combination with AMBP-1 has been shown to have protective effects on reducing the damage of various organs caused by a low oxygen and blood supply condition or ischemia in animal models, including gut [19], liver [20], kidneys [21], and brain [22]. These studies demonstrated the effectively therapeutic activity of AM/AMBP-1 which prompted us to evaluate the potential of applying AM/AMBP-1 in facilitating wound healing.

The inflammatory response begins shortly after wound injury with vasodilation, leading to increased blood flow to the site of injury together with an elevation of vascular permeability. AM is a potent vasodilator and AMBP-1 potentiates AM-induced vascular relaxation under normal and pathological conditions [23,24]. In this study, we used a rat model of cutaneous wound to assess the effect of topical AM and AMBP-1 combined treatment (AM/AMBP-1) on the healing process. We first compared the closure rate of wound between AM/AMBP-1- and vehicle-treated animals. We then examined the expression of several molecules involved in inflammation, angiogenesis and wound remodeling at different time frames of the healing process.

## Materials and Methods

### Experimental animals

Male adult (3–4 months) Sprague-Dawley rats (250–300 g) were purchased from Charles River Laboratories (Wilmington, MA). The rats were housed in a temperature controlled room and on a 12-h light/dark cycle. The rats were fed a standard Purina rat chow diet and allowed water *ad libitum*. Animal experimentation was carried out in strict accordance with the Guide for the Care and Use of Laboratory Animals of the National Institutes of Health. The protocol was approved by the Institutional Animal Care and Use Committee at the Feinstein Institute for Medical Research. All surgery was performed under isoflurane anesthesia, and all efforts were made to minimize suffering.

### Rat model of dorsal cutaneous wound

Prior to surgery, rats were fasted overnight, but water was given *ad libitum*. Rats were anesthetized with isoflurane inhalation. Circular full thickness cutaneous wounds were created on the dorsum. Briefly, the dorsum was shaved, followed by a 10% povidone-iodine wash. One circular 2.0 cm full thickness wound was immediately created on the dorsum using a trephine. The wounds were extended to the muscular layer excluding the panniculus carnosus. Bleeding was stopped by compression with cotton sticks. The animals were randomly divided into vehicle and AM/AMBP-1 treatment groups.

### AM/AMBP-1 preparation and administration

Human AM was purchased from Phoenix Pharmaceuticals (Belmont, CA). AMBP-1 was purified from human plasma as previously described [25]. Immediately after surgery, the rats were given a subcutaneous injection of buprenorphine (0.05 mg/kg BW) for post-operative analgesia and were resuscitated intraperitoneally with 10 ml/kg BW of 0.9% saline. The wounds were immediately covered with hydrofiber dressing containing AM/AMBP-1 (96/320 μg) or normal saline as vehicle. The dressings were kept in place by a modified rat jacket and were changed daily. Rat AM is similar to human AM with 50 amino acid residues, with two amino acid deletions and six substitutions from human AM [26]. We have reported studies with human AM in combination with human AMBP-1 in several inflammatory disease models such as sepsis [27], hemorrhagic shock [28] ischemia reperfusion injury [29] [20] and focal cerebral ischemia [22]. The ratio of AM to AMBP-1 is chosen based on prior studies [19–22].

### Measurement of wound size

The residual wound area was traced on a transparent film every other day after skin excision until day 14 and the pixel of the traced area was analyzed by NIH ImageJ. Wound closure rate was calculated according to the following formula: Wound closure rate (%) = [(Area_day 0_ - Area_day n_) /Area_day 0_] × 100; where Area_day 0_ is the initial wound area at day 0 and the Area_day n_ is the area on day n after wounding.

### Real-time polymerase chain reaction (PCR) analysis

Total RNA was extracted from skin tissues by using the Trizol reagent (Invitrogen, Carlsbad, CA) and reverse-transcribed into cDNA by using murine leukemia virus reverse transcriptase (Applied Biosystems, Foster City, CA). A PCR reaction was carried out in a 25 μl of final volume containing 0.08 μmol of each forward and reverse primer, cDNA, and 12.5 μl SYBR Green PCR Master Mix (Applied Biosystems). Amplification was conducted in an Applied Biosystems 7300 real-time PCR machine under the thermal profile of 50°C for 2 min, 95°C for 10 min and followed by 45 cycles of 95°C for 15 sec and 60°C for 1 min. The level of rat β-actin mRNA was used for normalization. Relative expression of mRNA was expressed as fold change in comparison to skin tissues without wound. The primers used for the gene expression profile are listed in Table 1. At each indicated day, the wounded skin tissues included the scab and epithelial margins were excised from the rat. Each rat had only one wound. The harvested tissues were cut through the central line into half. One section was fixed for histological analyses and the other was used for RNA extraction. The control samples were obtained from the skin of the rat without the wound and designated as day 0.

**Table 1:** A list of primer sequences used in this study.

Name	GenBank	Forward	Reverse
**TNF-α**	NM_012675	TGATCGGTCCCAACAAGGA	GGGCCATGGAACTGATGAGA
**IL-6**	NM_012589	AGGGAGATCTTGGAAATGAGAAAA	CATCATCGCTGTTCATACAATCAG
**VEGF**	NM_031836	CAGCTATTGCCGTCCAATTGA	CCAGGGCTTCATCATTGCA
**TGFβ-1**	NM_021578	GCGGACTACTACGCCAAAGA	TACCAAGGTAACGCCAGGAA
**Procollagen-I**	NM_001106366	CAGCGGCTACATCAACTCAA	AGAAGAGGCGAGTTCCTTCC
**Procollagen-III**	NM_178101	GTTGGCTGGAGGGTATGAGA	GAAGGTGGACGAGTCATGGT
**MMP-2**	NM_031054	ACCGTCGCCCATCATCAA	TTGCACTGCCAACTCTTTGTCT
**MMP-9**	NM_031055	TCGAAGGCGACCTCAAGTG	TTCGGTGTAGCTTTGGATCCA
**β-actin**	NM_031144	CGTGAAAAGATGACCCAGATCA	TGGTACGACCAGAGGCATACAG

### Histopathological and immunohistochemistry analysis

The wounded skin tissues were harvested and fixed in 10% formalin. Tissue blocks were cut in 4-μm sections, mounted on glass, followed by Masson-Trichrome (collagen) staining for light microscopy analysis. For immunostaining, paraffin-embedded sections were deparaffinized with xylene and dehydrated in decreasing concentrations of ethanol. Tissue sections were processed in 10 mM citrate buffer (pH 6.0), heated to 100°C for 10 min for antigen retrieval, and then treated with 3% hydrogen peroxide in methanol for 10 min to block endogenous peroxidase activity. Sections were incubated with primary antibodies (New England Biolabs) against anti-MPO, anti-CD31, anti-MMP-2, anti-Ki67 and anti-alpha smooth muscle actin (α-SMA; abcam), overnight at 4°C. The sections were incubated in an Envision system (DAKO, Carpinteria, CA) for 30 minutes at 37°C with washing in phosphate-buffered saline solution before incubation, diaminobenzidine solution was used as the color reagent.

### Statistical analysis

All data are expressed as means ± SEM (n = 4–5) and compared by Student’s *t*-test for two groups. Differences in value were considered significant if *P* < 0.05.

## Results

### AM/AMBP-1 accelerated wound closure

**Fig 1:**
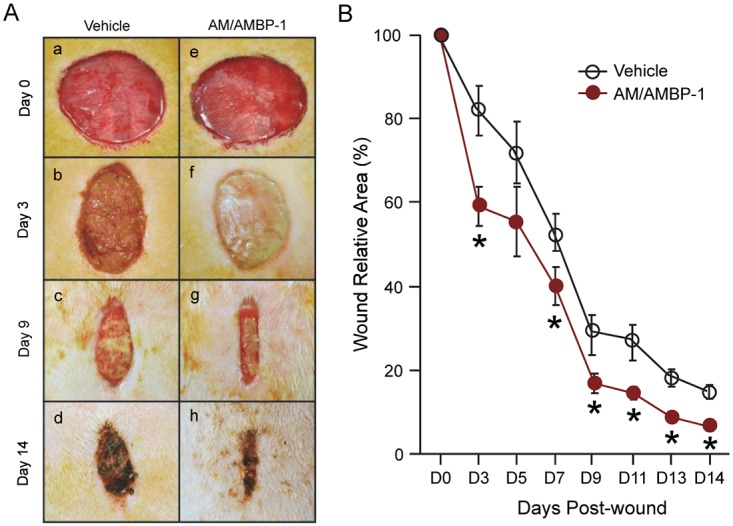
AM/AMBP-1 accelerated wound closure. Cutaneous wound created on rats were either treated with Vehicle or AM/AMBP-1 for 14 days and wound surface area were measured every other day. **A**. Photographical assessment of gross morphological changes observed during wound healing on different days of either vehicle treated (a-d) or AM/AMBP-1 treated (e-h) rats. **B**. Wound closure rate was assessed based on measuring wound size as described under Methods using NIH Image J. Data are represented as mean ± SEM (n = 5/group) and compared by Student’s *t*-test. *P<0.05 vs. Vehicle.

We first evaluated the effect of AM/AMBP-1 treatment on the healing rate of the cutaneous wound in rats. As shown in Fig. 1, topical application of AM/AMBP-1 significantly accelerated wound closure compared with vehicle throughout 14 days of observation. From day 3 to day 14, wound size was significantly reduced in the AM/AMBP-1 treatment group as compared to the corresponding vehicle group. Especially, at day 3 post-wound creation, AM/AMBP-1 presented a significant 42% reduction in wound size compared with vehicle (Fig. 1) indicating that daily treatment of AM/AMBP-1 accelerated cutaneous wound closure.

### AM/AMBP-1 promoted early resolution of wound-associated inflammation

**Fig 2:**
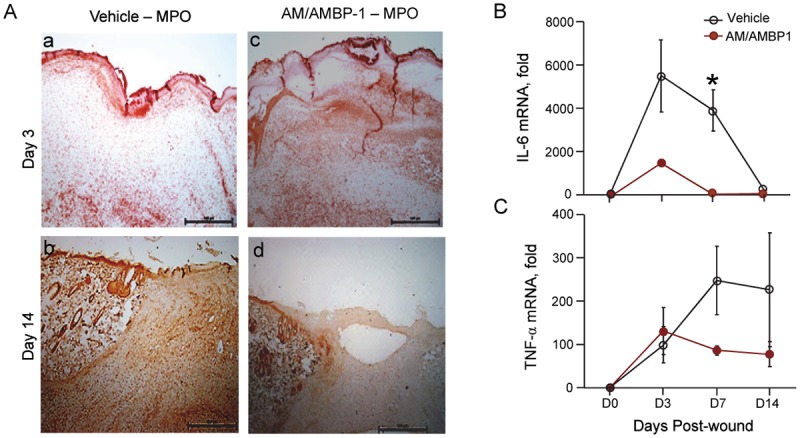
AM/AMBP-1 promoted resolution of inflammation after wound. **A**. Skin tissues from Vehicle (**a, b**) and AM/AMBP-1 (**c, d**) treated animals from day 3 (**a, c**) and day 14 (**b, d**) were sectioned and immunostained with myeloperoxidase (MPO) antibody. Total RNA extracted from skin tissues were analyzed by real time RT-PCR for IL-6 (**B**) and TNF-α (**C**) mRNA expressions. Data are represented as mean ± SEM (n = 4–5/group) and compared by Student’s *t*-test. *P<0.05 vs. Vehicle.

Neutrophil infiltration to the wound is required for clearance of cellular debris in preparation for the proliferative phase of wound healing. By immunostaining the wounded skin tissues with myeloperoxidase (MPO), a marker of activated neutrophils, we observed that at day 3, the areas with positive staining in AM/AMBP-1 treatment group were much more than those in the vehicle group (Fig. 2). In contrast at day 14, the areas with MPO positive staining in the vehicle group were more than those in the AM/AMBP-1 treatment group (Fig. 2). We further analyzed the inflammation status of the wounded skin by measuring gene expression levels of proinflammatory cytokines, IL-6 and TNF-α, using real time RT-PCR. The IL-6 levels in vehicle group were dramatically increased at days 3 and 7, and then returned to basal level at day 14 (Fig. 2). The IL-6 levels in AM/AMBP-1 treatment group were also elevated at day 3 but no statistical difference was observed between the two groups (Fig. 2). Interestingly, the IL-6 levels already dropped to the basal levels at day 7 with AM/AMBP-1 treatment and the values were significantly lower than that of vehicle group (Fig. 2). There were 4–5 animals each for different time points and groups. The expression of TNF-α in AM/AMBP-1 treatment and vehicle groups was increased to a similar level at day 3 (Fig. 2). While the TNF-α levels in vehicle group continued to increase at days 7 and 14, its level started to drop at day 7 and remained low at day 14 with AM/AMBP-1 treatment (Fig. 2). Taken together, AM/AMBP-1 treatment initially promoted neutrophil infiltration and activated proinflammatory cytokines during wound healing and attenuated subsequent neutrophil infiltration and facilitated resolution of inflammation induced by wound injury.

### AM/AMBP-1 shortened the angiogenesis phase

**Fig 3:**
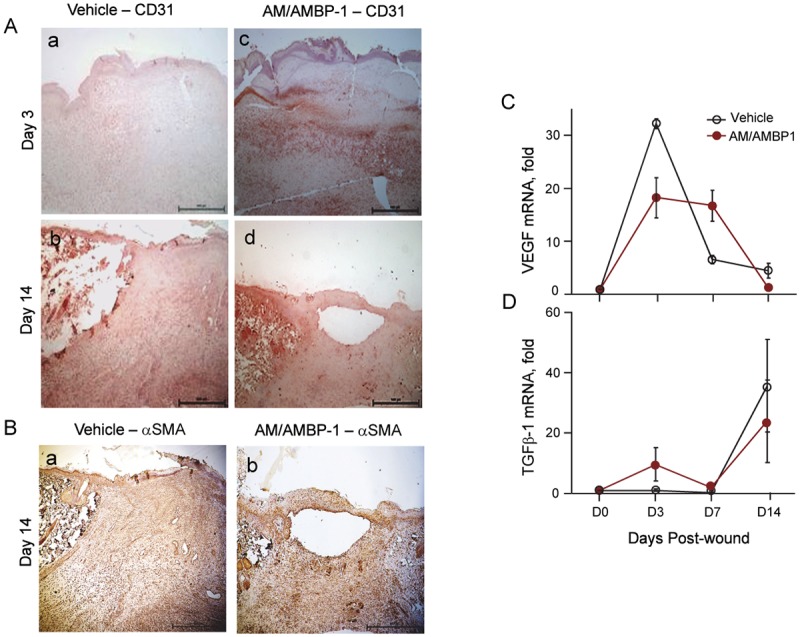
AM/AMBP-1 shortened angiogenesis phase during wound closure. **A**. Skin tissues from Vehicle (**a, b**) and AM/AMBP-1 (**c, d**) treated animals from day 3 (**a, c**) and day 14 (**b, d**) were sectioned and immunostained with CD31 antibody. **B**. Skin tissues from Vehicle (**a**) and AM/AMBP-1 (**b**) treated animals from day 14 were sectioned and immunostained with α-SMA antibody. Total RNA extracted from skin tissues were analyzed by real time RT-PCR for VEGF (**C**) and TGFβ-1 (**D**) mRNA expressions. Data are represented as mean ± SEM (n = 4–5/group) and compared by Student’s *t*-test. *P<0.05 vs. Vehicle.

After the inflammatory phase, the process of wound healing is moved to angiogenesis stage. We first observed that at day 3, AM/AMBP-1 treated cutaneous wounds had more staining of CD31, a marker of angiogenesis, than that in vehicle group (Fig. 3). However, at day 14, the AM/AMBP-1-treated wounds only had a few areas with CD31-staining and increased α-SMA expression (Fig. 3) indicating these wounds were already entering the maturation phase. In contrast, the wounds in vehicle group at day 14 still had a large area stained with CD31 and very low α-SMA expression (Fig. 3), indicating these wounds were still under active angiogenesis stage.

We then examined the gene expression of two important proangiogenic factors, VEGF and TGFβ-1, in skin tissues by real time RT-PCR. As shown in Fig. 3, VEGF expression was increased at day 3 in both AM/AMBP-1 treatment and vehicle groups, although the levels in vehicle group were higher than AM/AMBP-1 treatment group. However, at day 7, VEGF expression in the vehicle group was lower than that in AM/AMBP-1 treated wounds (Fig. 3). At day 14, VEGF expression in AM/AMBP-1 treatment group returned towards baseline, whereas the levels in the vehicle group were still 4.5-fold higher than the baseline levels (Fig. 3). There was a biphasic stimulation of TGFβ-1 expression in AM/AMBP-1 treatment group. The levels was increased to 9.7-fold at day 3, dropped to basal levels at day 7, and elevated again to 24-fold at day 14 (Fig. 3). However, a 36-fold increase of TGFβ-1 expression at day 14 was observed in the vehicle group (Fig. 3).

**Fig 4:**
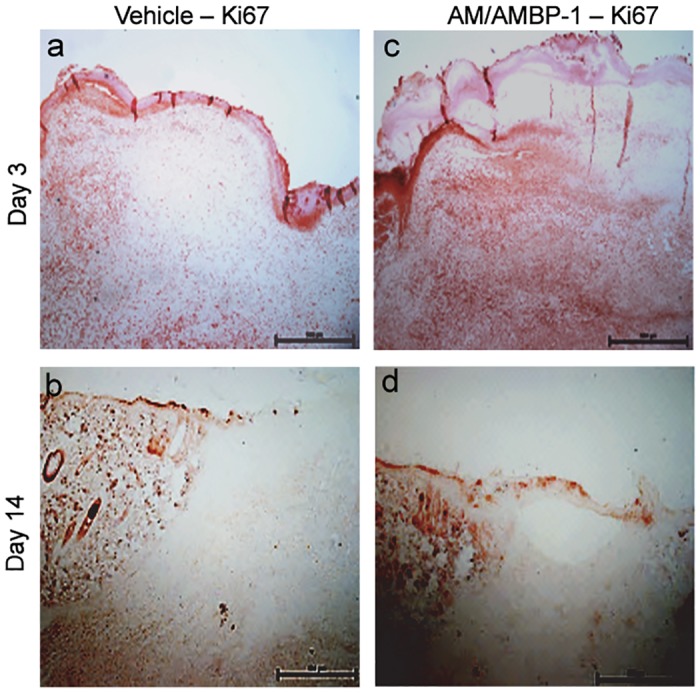
AM/AMBP-1 promoted wound maturation. **A**. Skin tissues from Vehicle (**a, b**) and AM/AMBP-1 (**c, d**) treated animals from day 3 (**a, c**) and day 14 (**b, d**) were sectioned and immunostained with Ki67 antibody.

The process of angiogenesis is characterized by proliferation of endothelial cells and smooth muscle cells. We assessed the cellular proliferation status during wound healing by performing immunohistochemistry in skin tissues for Ki-67, a proliferation marker. As shown in Fig. 4, there was more intense staining of Ki-67 in AM/AMBP-1-treated wounds than in vehicle wounds at day 3. When the wounds are toward to maturation, the activity of angiogenesis decreases and the cells in the wound area become less proliferating. Correspondingly, at day 14, AM/AMBP-1-treated wounds did not show much positive Ki-67 staining, while vehicle wounds still had some areas stained with Ki-67 (Fig. 4), indicating an early completion of wound maturation in AM/AMBP-1 treatment in comparison to the vehicle group.

### AM/AMBP-1 increased collagen deposition

**Fig 5:**
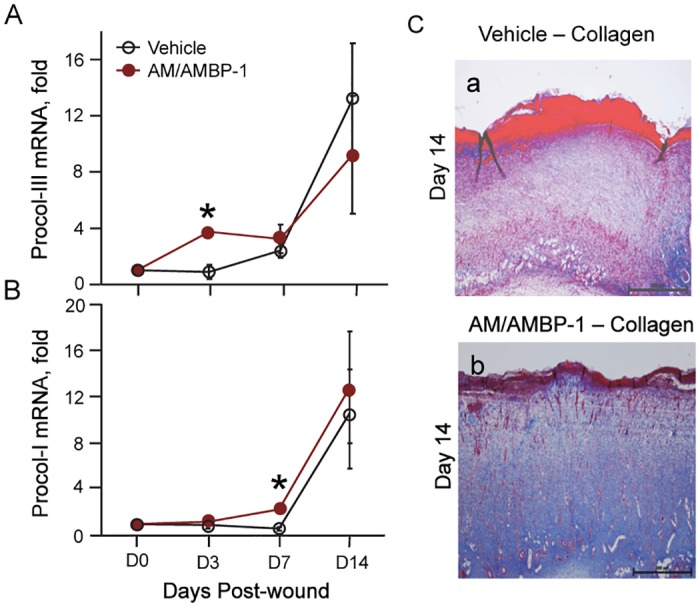
AM/AMBP-1 augmented collagen deposition. Total RNA extracted from skin tissues were analyzed by real time RT-PCR for Pro-collagen III (**A**) and Pro-collagen I (**B**) mRNA expressions. **C**. Skin tissues from Vehicle (**a**) and AM/AMBP-1 (**b**) treated animals on day 14 were stained with Masson trichrome for collagen deposition. Data are represented as mean ± SEM (n = 4–5/group) and compared by Student’s *t*-test. *P<0.05 vs. Vehicle.

During the proliferative phase of wound healing, fibroblasts migrate into the wound and secrete collagen. Fibroblast migration and collagen deposition together with angiogenesis forms granulation tissue. It has been shown that fibroblast accumulation in the wound is accompanied by an initial deposition of collagen-III, which is later replaced by collagen-I. We determined the gene expression of procollagen-III and -I in skin tissues. AM/AMBP-1 treatment markedly increased procollagen-III expression by 3.6-fold in comparison with vehicle at day 3 (Fig. 5). After day 7, procollagen-I expression started elevating in both AM/AMBP-1 treatment and vehicle groups (Fig. 5). By performing Masson-Trichrome staining in the skin tissues, we observed that the overall collagen content in the AM/AMBP-1 treated wounds (Fig. 5) was much higher than that in the vehicle wounds at day 14 (Fig. 5).

### AM/AMBP-1 enhanced MMP- 2 and MMP-9 expression

**Fig 6:**
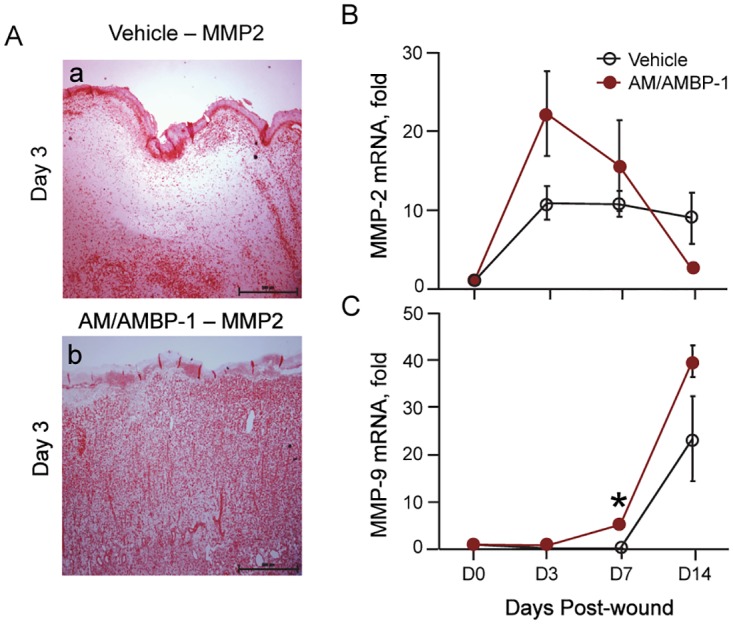
AM/AMBP-1 enhanced MMP-2 and MMP-9 expressions. **A**. Skin tissues from Vehicle (**a**) and AM/AMBP-1 (**b**) treated animals on day 3 were immunostained with MMP-2 antibody. Total RNA extracted from skin tissues were analyzed by real time RT-PCR for MMP-2 (**B**) and MMP-9 (**C**) mRNA expressions. Data are represented as mean ± SEM (n = 4–5/group) and compared by Student’s *t*-test. *P<0.05 vs. Vehicle.

The activation of matrix metalloproteinases are required for extracellular matrix remodeling during wound healing. By conducting an immunohistochemical staining of MMP-2 in skin tissues, we observed that the MMP-2 positive areas in AM/AMBP-1-treated wounds were more than those in vehicle wounds at day 3 (Fig. 6). Correspondingly, gene expression levels of MMP-2 in the AM/AMBP-1-treated wounds were higher than those in vehicle group at days 3 and 7 (Fig. 6). By day 14, AM/AMBP-1-treated wounds showed a decline of MMP-2 gene expression to the basal levels, while its level in vehicle wounds were still 8.6-fold higher than the basal level (Fig. 6). Meanwhile, MMP-9 expression started to increase at day 7 and remained high levels at day 14 in both AM/AMBP-1 treatment and vehicle groups, although the levels in the AM/AMBP-1 treatment group were slightly higher than those in the vehicle groups (Fig. 6).

## Discussion

Using a full-thickness cutaneous wound model, we demonstrated for the first time that topical administration of AM/AMBP-1 given daily for 14 days accelerated cutaneous wound healing as early as day 3. AM/AMBP-1 promoted all three phases of wound healing, leading to early wound closure and maturation. AM/AMBP-1 treatment promoted early infiltration of the wound by neutrophils, which are important in the early phase of wound healing. The neutrophils migrate across the endothelium by paracellular or transendothelial routes, a process mediated by molecules such as intercellular adhesion molecule 1 (ICAM-1) and platelet/endothelial-cell adhesion molecule 1 [30]. Once in the wound, neutrophils play an essential role in phagocytosing tissue debris. However, persistent neutrophil infiltration could exacerbate tissue injury by the release of toxic mediators such as reactive oxygen species, leading to delayed wound healing [30]. We showed that effect of AM/AMBP-1 on neutrophil infiltration was time-dependent; causing a beneficial early increase in wound neutrophils followed by a more rapid neutrophil depletion.

We also demonstrated that AM/AMBP-1 accelerated angiogenesis in cutaneous wounds via the upregulation of proangiogenic factors, VEGF and TGFβ-1. Correspondingly, on immunhistochemical staining, the expression levels of the angiogenesis marker, CD31, were increased after AM/AMBP-1 treatment during early stages of the wound and decreased at the matured stage. The decrease in CD31 expression was correlated with an increase in α-SMA expression indicating the presence of mature blood vessels. Angiogenesis during wound healing is accompanied by fibroblasts migration into the wound and subsequent collagen deposition [30]. We demonstrated by Ki-67 staining that overall cellular proliferation in cutaneous wounds was increased by AM/AMBP-1 treatment. Our findings are supported by previous studies which reported that AM promotes endothelial cell, smooth muscle cell and fibroblast proliferation [31,32]. Our study also revealed that AM/AMBP-1 hastened wound maturation. During wound healing, collagen-III is initially deposited by fibroblasts. As the wound matures, collagen-III is replaced by collagen-I [9,10]. We demonstrated that AM/AMBP-1 treatment significantly upregulated pro-collagen-III compared with vehicle at day 3. At day 7, AM/AMBP-1 treated wounds were found to have higher expression of pro-collagen-I compared with vehicle, indicating wound maturation.

Matrix metalloproteinases (MMPs) and growth factors are also known to be involved in the wound healing process [30]. MMP-2 and MMP-9 are key players during remodeling and reepithelialization [30]. As part of wound maturation, the collagen matrix undergoes remodeling through the activity of matrix metalloproteinases [30]. We showed that AM/AMBP-1 increased the expression of MMP-2 and MMP-9 mRNA in the cutaneous wounds at days 3 and 7, respectively. MMP-2 expression began to decline after day 7, almost reaching baseline levels by day 14 in AM/AMBP-1 treated wounds, while the expression levels in vehicle treated wounds continued to plateau at day 14. This suggests that AM/AMBP-1 treatment alters the temporal expression between MMP-2 and MMP-9 to accelerate the completion of tissue remodeling after the wound. Chronic wounds are also associated with an exaggerated inflammatory response and a major cause for delayed wound healing. Proinflammatory cytokines (TNF-α, IL-1, IFN-γ) and MMP-9 are elevated in chronic wound fluids [33–37]. We showed that while both TNF-α and IL-6 mRNAs continued to increase in the vehicle group, with AM/AMBP-1 treatment their levels increased by day 3 but lowered by day 7 and day 14 indicating resolution of inflammation. Therefore, therapeutic targeting as early as the inflammatory stage could accelerate the wound healing process and produce efficient wound closure.

Contraction is one of the major features of healing in rat wounds [38–40]. Contraction is mediated by myofibroblasts which are characterized as muscle like cells that interact with the extracellular matrix such as collagen. Unlike humans, rats are loose-skinned animals with minimal adherence to the underlying structures. The skin of loose-skinned rats can retract easily after an incision creating a large gap. Wound contraction which is more rapid than epithelialization causes the skin to close and decrease the wound healing time. Contraction can be measured by simply tracing the wound every other day and represent as a percent change in the wound surface area compared to the original wound size. In full thickness wound as in the current study, the epithelialization comes from the wound edge and occurs at a rate of 1–2 mm/day and seems to occur in 24–48 h [41]. In this regard, in the representative figure shown in Fig. 1A appears that the original wound size on day 0 in the AM/AMBP-1 is larger than that of vehicle. The possible cause for this discrepancy is the retraction of the wound due to the loose-skin creating uneven gap. However, on day 3, the percent change from the original size on day 0 in AM/AMBP-1 was significantly higher than the percent change in vehicle. One possibility for this observation is wound contraction which is a major disadvantage of the rat wound healing model. However, the likely possibility is that the epithelialization in AM/AMBP-1 group was accelerated in comparison to the vehicle group.

In summary, we have demonstrated the therapeutic potential of AM/AMBP-1 in wound healing. AM/AMBP-1 accelerated wound healing by promoting angiogenesis, fibroblasts migration, collagen deposition and wound remodeling. AM/AMBP-1 may be further developed as a novel therapy for cutaneous wounds.
